# Molecular and phylogenetic analysis of HIV-1 variants circulating in Italy

**DOI:** 10.1186/1750-9378-3-13

**Published:** 2008-10-10

**Authors:** Luigi Buonaguro, Annacarmen Petrizzo, Maria Tagliamonte, Francesca Vitone, Maria Carla Re, Elisabetta Pilotti, Claudio Casoli, Costanza Sbreglia, Oreste Perrella, Maria Lina Tornesello, Franco M Buonaguro

**Affiliations:** 1 Laboratory of Molecular Biology and Viral Oncogenesis & AIDS Reference Center, Istituto Nazionale Tumori "Fondazione Giovanni Pascale", Naples – Italy; 2 Section of Microbiology of the Department of Hematology, Oncologic Science, Anatomical Pathology and Microbiology, University of Bologna, Bologna – Italy; 3 Interuniversity Consortium, National Institute Biostructure and Biosystem (INBB), Rome – Italy; 4 Department of Clinical Medicine, Nephrology, and Health Sciences, University of Parma, Parma – Italy; 5 Department of Clinical Sciences, Infectious Diseases Unit 'L. Sacco', University of Milano – Italy; 6 VII Division of Infectious Diseases, Cotugno Hospital, Naples – Italy

## Abstract

**Objective:**

The continuous identification of HIV-1 non-B subtypes and recombinant forms in Italy indicates the need of constant molecular epidemiology survey of genetic forms circulating and transmitted in the resident population.

**Methods:**

The distribution of HIV-1 subtypes has been evaluated in 25 seropositive individuals residing in Italy, most of whom were infected through a sexual route during the 1995–2005 period. Each sample has been characterized by detailed molecular and phylogenetic analyses.

**Results:**

18 of the 25 samples were positive at HIV-1 PCR amplification. Three samples showed a nucleotide divergence compatible with a non-B subtype classification. The phylogenetic analysis, performed on both HIV-1 *env *and *gag *regions, confirms the molecular sub-typing prediction, given that 1 sample falls into the C subtype and 2 into the G subtype. The B subtype isolates show high levels of *intra*-subtype nucleotide divergence, compatible with a long-lasting epidemic and a progressive HIV-1 molecular diversification.

**Conclusion:**

The Italian HIV-1 epidemic is still mostly attributable to the B subtype, regardless the transmission route, which shows an increasing nucleotide heterogeneity. Heterosexual transmission and the interracial blending, however, are slowly introducing novel HIV-1 subtypes. Therefore, a molecular monitoring is needed to follow the constant evolution of the HIV-1 epidemic.

## Introduction

Human immunodeficiency virus type 1 (HIV-1) shows an extensive genetic variability and can be classified into 9 phylogenetic subtypes (A-K), which are approximately equidistant from one another, and several circulating recombinant forms (CRFs), resulting from recombination events occurring between different HIV-1 subtypes co-circulating in a specific geographic region [1].

The first phase of the HIV epidemic in Italy has been mainly confined to the injecting drug users (IDUs) risk group, with an absolute predominance of HIV-1 B subtype, in accordance with other Western Countries. In particular, among the total AIDS cases reported in the adult population during the period between 1982 and 2006, 56.0% were IDUs (including also homosexual IDUs) with similar percentages in men and women groups (57.3% and 51.5%, respectively) [2]. The annual percentages of AIDS cases reported in IDUs have gradually decreased from 65.8% in 1987 to 27.6% in 2006 [2], in part as consequence of prevention programs implemented in Italy to discourage syringe sharing [3,4]. In parallel, the overall AIDS cases reported in heterosexual individuals account for the 19.5% of total epidemic cases, with a significantly higher percentage in the women category compared to men (41.2% vs 13.6%). However, the annual percentage of AIDS cases related to the heterosexual transmission has dramatically increased over the years, becoming in 2006 the most prevalent risk factor for AIDS (40.4%) [2].

Although almost 25% of heterosexual individuals diagnosed with AIDS in Italy are partners of long-term HIV-1 infected individuals, carrying a "historical" B-subtype virus, more than 10% of them are either immigrants from endemic regions for HIV-1 (6.87%) or their Italian partners (3.03%), while the risk is unidentified for 64% of them [2]. This epidemiological evidence, based only on the AIDS reported cases and not considering all the HIV-1 infections derived also from travelling abroad, suggests that at least 10% of the viruses transmitted through heterosexual contacts could potentially belong to non-B subtypes and CRFs. In fact, HIV-1 isolates genetically related to subtypes novel to the Italian epidemic have been recently increasingly identified and described [5-17].

This has been recently reported in other European Countries, with a higher prevalence of non-B subtypes and CRFs due to an older tradition of immigration waves and much tighter historical as well as economic links with countries endemic for HIV-1 infection [18-30].

In this framework, the introduction and the possible spread of different HIV-1 subtypes and/or recombinant forms, which could require the future development of adequate diagnostic, treatment, and prevention strategies, needs to be constantly monitored.

For the present study, 25 HIV-seropositive individuals residing in Italy were enrolled at sentinel Centers, with a HIV-infection diagnosed in the period 1995–2005. The molecular study has been performed on the hypervariable C2-V3 region of the *env *gene as well as the more conserved 5' region of the *gag *p17 sequence. Three non-B-subtype HIV-1 isolates have been identified and phylogenetically classified as C (1 isolate) and G (2 isolates) subtype.

## Methods

### Sample Collection

Blood samples were collected from 25 HIV-positive individuals attending Italian sentinel Centers in Bologna, Parma and Naples. For all of them, HIV infection was diagnosed during the 1995 to 2005 period, and most participants were infected through sexual contact (18 of 25, 72%). HIV-1 infection was diagnosed by immunologic methods (ELISA, Western blot), and the viral load was evaluated by viral RNA quantification. The full designation of samples, according to WHO-proposed nomenclature, is CI05.00XE or CI05.00XG, where CI stands for the city of enrolment, 05 stands for the year of study, 00 for the enrolment number and E (or G) stands for *env *(or *gag*). For the sake of simplicity, however, in this paper the samples have been indicated with CI05.01 (i.e. BO05.01).

### Polymerase Chain Reaction (PCR)

Peripheral blood mononuclear cells (PBMCs) were purified from fresh HIV-1-positive blood samples by Leucoprep density gradient centrifugation, and cellular lysates (approximately 6 × 10^6 ^cells) were prepared by Proteinase K digestion at 56°C.

The quality of target DNA was verified by PCR amplification of the housekeeping p53 cellular gene. The amplification of HIV-1 V3-V5 region of the *env *gene and p17 region of the *gag *gene were performed by nested PCR analysis, using 1.5 × 10^5 ^cells (corresponding to approximately 1 μg of genomic DNA) as a template. The V3-V5 region of the HIV-1 *env *gene (666 bp) was amplified, as previously described, using the primer pairs ED5-ED12 and ES7-ES8 for the first and the second round of amplification, respectively [31,32]. The p17 region (474 bp) was amplified, as previously described, using the primer pairs CL1028-CL1033 and CL1029-CL1032 for the first and the second round of amplification, respectively [14,33].

### DNA Sequencing

Direct sequencing reactions were performed on PCR products purified with a rapid method developed in our laboratory, following the Sequenase protocol (United States Biochemical, Cleveland OH), modified in the labelling step (3 minutes on ice) [31,34]. The internal annealing oligonucleotides, V3B and GAG B SENSE (annealing to a 19 bp fragment of the C2-V3 region and a 17 bp of the p17 region, respectively), were used to prime sense sequence reactions. Sequences were then analyzed on 6% polyacrylamide wedge sequencing gel.

### Analysis of Sequences

The *env *and *gag *sequences obtained were aligned. Multiple sequence alignments were performed with the MegAlign application of the Lasergene software (DNASTAR Inc., Madison, WI) using the Clustal method. Phylogenetic trees were generated by using the neighbor-joining method with the PHYLIP software package (version 3.52c; Joseph Felsenstein, University of Washington). Briefly, the SEQBOOT program was carried out to generate 100 data sets that represent randomly re-sampled versions of the input-aligned sequences, to test the reliability of the final tree topology. Evolutionary distances were estimated by the DNADIST program, using either the Kimura 2-parameter method or the maximum likelihood distance method, and the phylogenetic relationships were determined by the NEIGHBOR program. A consensus tree was constructed using the CONSENSE program with the majority rule criterion and was drawn with the NJPLOT application.

## Results

### Epidemiologic and clinical parameters

Overall, 25 HIV-1-seropositive subjects were enrolled in the study. Of them, 32% (8 of 25) were heterosexuals, 36% (9 of 25) were male homosexuals, 4% (1 of 25) was a bisexual and 28% (7 of 25) were IDUs. All but 6 subjects were native Italian, with stable or occasional sexual partners of different nationalities. In particular, the enrolled subjects included an Ethiopian (PR06.03), an Albanian (NA05.04), an Ivorian (PR06.05), a Venezuelan (PR06.06) and two Nigerians (NA05.03, PR06.07). The CD4+ counts ranged from 169 to 1387 cells/mm^3 ^(median, 664 cells/mm^3^) (Table 1).

**Table 1:** Epidemiologic and clinical parameters of enrolled subjects.

**Sample Code**	**Nationality**	**Risk Factor**	**Presumed infection date**	**CD4+/μl**
**BO07.01**	Italian	HOMOSEX	2002	886

**BO07.06**	Italian	IDU	1998	1110

**BO07.09**	Italian	HETEROSEX	1997	533

**BO07.12**	Italian	HOMOSEX	1999	515

**BO07.13**	Italian	IDU	1995	718

**BO07.14**	Italian	IDU	1998	659

**BO07.18**	Italian	HOMOSEX	2001	1387

**BO07.20**	Italian	IDU	2000	515

**BO07.23**	Italian	HETEROSEX	2002	765

**BO07.24**	Italian	IDU	2003	917

**BO07.25**	Italian	HOMOSEX	2001	462

**BO07.26**	Italian	IDU	2000	279

**NA05.01**	Italian	IDU	1999	664

**NA05.02**	Italian	HOMOSEX	2000	427

**NA05.03**	Nigerian	HETEROSEX	2002	1203

**NA05.04**	Albanian	HETEROSEX	2003	391

**NA05.05**	Italian	HETEROSEX	2005	898

**NA05.06**	Italian	HOMOSEX	2005	459

**PR06.01**	Italian	HOMOSEX	2001	764

**PR06.02**	Italian	BISEX	1999	169

**PR06.03**	Ethiopic	HETEROSEX	2002	481

**PR06.04**	Italian	HOMOSEX	2000	852

**PR06.05**	Ivorian	HETEROSEX	1998	1100

**PR06.06**	Venezuelan	HOMOSEX	2001	1244

**PR06.07**	Nigerian	HETEROSEX	2001	389

### Amplification of Italian samples by PCR

**Figure 1:**
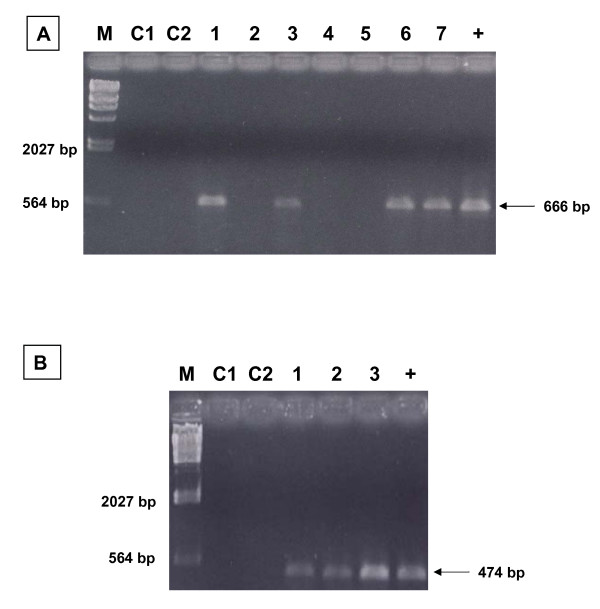
**Analysis of DNA fragments obtained by nested PCR on the C2-V5 *env *(A) and the p17 *gag *(B) genes of HIV-1**. Examples of specific *env* fragments of 666 bp (panel A) and *gag* fragments of 474 bp (panel B), obtained by nested PCR have been separated on a 1% agarose gel. M = λ/Hind III, C1 = I round negative control; C2 = II round negative control; + = positive control.

The C2-V5 region of the HIV-1 *env *gene and the p17 region of the *gag *gene were amplified by nested PCR using primers and conditions previously described [14]. Overall, 60% of the samples (15 of 25) were positive at the PCR amplification reactions for both *env *and *gag *sub-genomic regions. However, 64% of the samples (16 of 25) were positive at the amplification reaction for *env *and 68% of the samples (17 of 25) were positive to the amplification reaction for *gag*. Therefore, one sample (BO07.14) was positive only in *env *and two samples (NA05.05 e NA05.06) were positive only in *gag*; the remaining 7 samples were negative to both sub-genomic regions. This result might be the consequence of either mutations in the primers' annealing regions, reducing the melting temperature, or proviral DNA quantity below the threshold of sensibility. An example of the results obtained by nested PCR for *env *(666 bp) and *gag *(474 bp) is shown in Figure 1A and 1B.

### Molecular analysis of *env *gene

**Figure 2:**
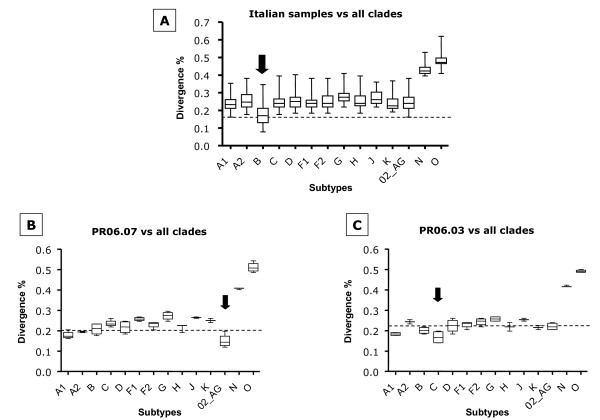
**Average nucleotide divergence of C2-V5 *env *gene versus standard sequences of different clades**. The shown average divergence values have been obtained aligning the Italian sequences with standard sequences of individual clades, Groups and CRF02_AG. (A) The lowest average divergence value of the whole group of sequences is versus B standard sequences (arrow); (B) the sample PR06.07 shows the lowest average divergence value versus CRF02_AG standards (arrow); (C) the sample PR06.03 shows the lowest average divergence versus clade C standard sequences (arrow) (p < 0.01).

The nucleotide sequence analysis has been performed on the C2-V3 region of the *env *gene, directly from the PCR products without a sub-cloning step. The Italian HIV-1 nucleotide sequences, aligned with HIV-1 reference standards of different subtypes, Groups and CRF02_AG, have been aligned with Clustal method, to determine the homology values between the analyzed samples. The results, show an average divergence of 19.66% within the Italian group, with values ranging from 7.1 to 33.9% (data not shown). The Italian sequences, showed average divergences of 17.97% versus standards of B clade; 25.87% versus standards of other HIV genotypes of Group M; 43.14% and 48.4% versus standards of Groups N and O, respectively (Figure 2A). Nevertheless, specific samples show a >30% divergence versus the B clade, strongly suggesting a non-B clade classification. In particular, the sample PR06.07 shows an average divergence of 15% versus the CRF02_AG and 23.2% versus all other clades (Figure 2B); the sample PR06.03 shows an average divergence of 18% versus the C clade and 22.7% versus all other clades (Figure 2C). Therefore, the results suggest a B-clade classification for the vast majority of the analyzed Italian sequences and a C-clade and CRF02_AG classification for the latter samples.

### Molecular analysis of *gag *gene

**Figure 3:**
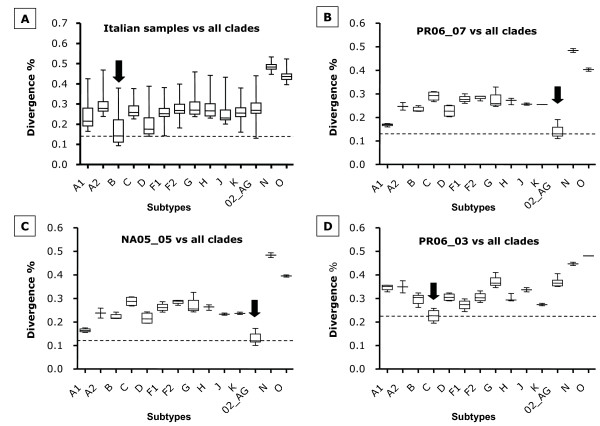
**Average nucleotide divergence of p17 *gag *gene versus standard sequences of different clades**. The shown average divergence values have been obtained aligning the Italian sequences with standard sequences of individual clades. (A) The lowest average divergence value of the whole group of sequences is versus B standard sequences (arrow); (B) the sample PR06.07 and (C) the sample NA05.05 show the lowest average divergence value versus CRF02_AG standards (arrow); (D) the sample PR06.03 shows the lowest average divergence versus clade C standard sequences (arrow) (p < 0.01).

The nucleotide sequence analysis has been performed on the p17 region of the *gag *gene, directly from the PCR products without a sub-cloning step. As for the *env *gene, the HIV-1 nucleotide sequences have been aligned with Clustal method to HIV-1 reference standards of different subtypes, Groups and CRF02_AG, in order to determine the homology values between the analyzed samples. The results show an average divergence of 15.86% within the Italian group, with values ranging from 10.81 to 29.16% (data not shown). The Italian sequences, showed average divergences of 16.88% versus standards of B clade; 25.99% versus standards of other HIV genotypes of Group M; 48.54% and 44.15% versus the standards of Groups N and O, respectively (Figure 3A). Nevertheless, specific samples show a >22% divergence versus the B clade, strongly suggesting a non-B clade classification. In particular, the sample PR06.07, also in the *gag *region, shows an average divergence of 13% versus the CRF02_AG and 24.34% versus all other clades (Figure 3B). Similarly, the sample PR06.03, also in the *gag *region, shows an average divergence of 22% versus the C clade and 32.08% versus all other clades (Figure 3D). Finally, the sample NA05.05, negative for *env*, shows an average divergence of 13.9% versus the CRF02_AG and 25.31% versus all other clades (Figure 3C). Therefore, also in the *gag *region, the results suggest a B-clade classification for the vast majority of the analyzed Italian sequences and confirm a C-clade or CRF02_AG classification for the same samples identified in *env*.

### Phylogenetic analysis of C2-V3 env and p17 *gag *sequences

Nucleotide sequences have been pairwise aligned with HIV-1 reference standards of different subtypes and CRFs, discounting gaps due to nucleotide insertions/deletions. The alignments of HIV-1 *env *and *gag *nucleotide sequences have been used to generate phylogenetic trees by the neighbor-joining method. Confidence values for individual branches have been determined by a bootstrap analysis. The reference standards of different subtypes as well as CRFs included in this study cluster in the expected distinct phylogenetic branches in both *env *and *gag *sub-genomic regions, indicating that the length of sequence fragments used in this analysis is sufficient for the identification of known subtypes.

**Figure 4:**
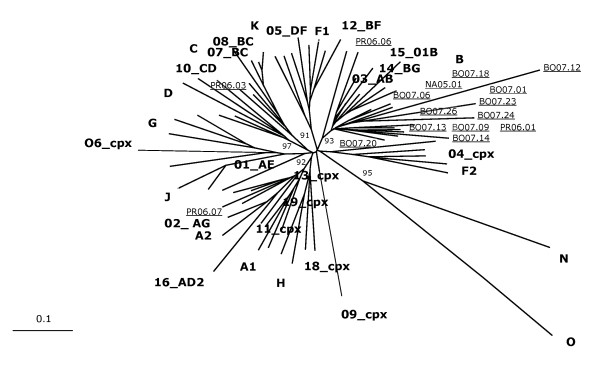
**Phylogenetic classification of the C2-V3 *env *region**. The C2-V3 *env *region of Italian samples has been aligned with standard sequences of HIV-1 group M including some known CRF. Sequences from groups N and O have been used as outgroup. Italian sequences are indicated as underlined. Reliability has been estimated by boot-strap analysis. The bar shows a 10% divergence.

**Figure 5:**
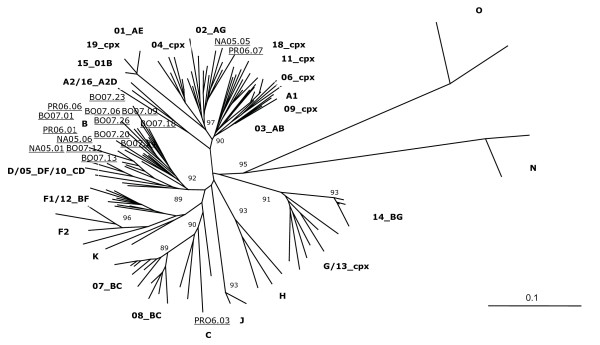
**Phylogenetic classification of the p17 region of *gag *gene**. The p17 region of *gag *gene from Italian samples has been aligned with standard sequences of HIV-1 group M including some known CRF. Sequences from groups N and O have been used as outgroup. Italian sequences are indicated as underlined. Reliability has been estimated by boot-strap analysis. The bar shows a 10% divergence.

All the Italian HIV-1 isolates showing a rate of nucleotide divergence less than 30% in *env *and 20% in *gag *versus standards of the B clade, phylogenetically cluster with reference standards of this clade in both *env *and *gag *sub-genomic regions, forming several sub-clusters. Similarly, the 3 samples showing a lower nucleotide divergence both in *env *and *gag *versus standards of the C clade or the CRF02-AG, cluster with these non-B subtype standards in both sub-genomic regions, confirming the tentative subtype classification based on nucleotide divergence values (Figure 4 and 5).

Moreover, all isolates show a concordant subtype classification in both *env *and *gag *sub-genomic regions, suggesting the absence of intra-genomic recombination events.

### Peptide analysis and comparison

**Figure 6:**
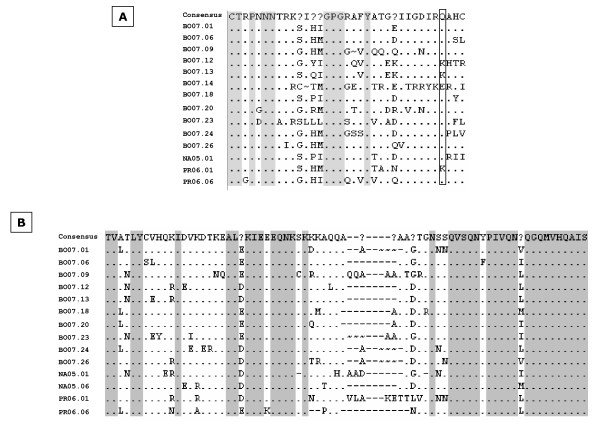
**Alignment of the amino acid sequences**. (A) V3 env region and (B) p17 gag region have been aligned, and a consensus sequence (top) has been generated. Question marks (?) have been introduced in the consensus when, in the specific position, no residue is found in more than 50% of sequences. Dots (.) indicate agreement with the consensus and dashes (-) indicate a gap inserted to maintain the alignment. Gray areas indicate perfectly conserved residues. Boxed residues indicate mutations possibly conferring resistance to class of drugs.

The V3 and p17 region amino acid sequences have been deduced for each isolate by computer analysis and aligned following their subtype classification. The V3 region of the Italian B-subtype isolates identified in this study shows an amino acid variability mainly localized outside the V3 loop region. The consensus derived from the alignment, in fact, shows that the amino acid residues conserved in 100% of the aligned sequences are all localized in the V3 loop (9 of 35, 25.71%). However, besides the subtype-specific "fingerprint" GPGR sequence at the tip of the V3 loop, less-represented sequences have been identified at the tip (GPGG, GPGS, GPGQ), suggesting a constant diversification in the B clade (Figure 6A).

In regards to the non-B clade isolates identified in this study, and distributed in two different clades (C and CRF02-AG), the too limited number of samples hampers the possibility to derive a consensus sequence and to infer conclusions; nevertheless, it is worthwhile to mention that they all show the "fingerprint" GPGQ tetramer at the tip of their V3 loop sequences (data not shown).

Moreover, mutations in amino acid residues conferring resistance to fusion/binding inhibitors, in association with other mutations along the env sequence not analyzed in the present study, have been observed (i.e. Q296K), suggesting the transmission of isolates resistant to this class of antiretrovirals (Figure 6A) [35-37].

The p17 region of the B clade isolates shows a low amino-acidic variability and the consensus derived from the alignment shows an overall rate of amino acid conservation of 44.44% (36 of 81 residues). None of the observed amino acid changes is found in gag residues conferring drug resistance (Figure 6B). Similar results are observed also for non-B clade isolates.

## Discussion

A molecular and phylogenetic characterization was performed on HIV-1 variants identified in individuals residing in Italy and infected in the 1995–2005 period. The groups at high risk of HIV-1 infection (heterosexuals, homosexuals and IDUs) were equally represented in the present study. The C2-V5 and p17 regions of the *env *and *gag *gene have been amplified from uncultured PBMCs of Italian samples by the standardized nested PCR conditions.

Informative regions of the 2 structural *gag *and *env *genes have been studied in parallel, to have an immediate picture of the evolution pattern in viral regions under different immune pressure and to identify possible intra-genomic recombinants. In fact, considering the worldwide represented CRFs described until now, the p17 *gag *and the C2-V3 *env *regions show a different subtype designation in most of cases and are highly diagnostic for the identification of novel recombinants.

The average nucleotide divergences versus standard sequences of different subtypes suggest the presence of B and non-B subtypes among the Italian sequences identified in the present study. Moreover, within the B subtype sequences, the average divergence in the env C2-V3 region is 18.08%, with values ranging from 7.1 to 29.9%. On the contrary, the average divergence in the gag p17 region is 11.13%, with values ranging from 7.79 to 16.86%. These observed divergence values are compatible with samples identified in a geographical area characterized by a long-lasting HIV epidemic and confirm the more pronounced genetic evolution of *env *gene compared to *gag*.

The phylogenetic analysis confirmed the nucleotide divergence subtype prediction for both B and non-B-subtype classification; in particular, of the 3 non-B isolates, 2 cluster with CRF02-AG (NA05.05 and PR06.07), 1 with the C subtype (PR06.03). Moreover, for all the B- and non-B-subtype sequences, the phylogenetic classification matches in the *gag *and *env *sub-genomic regions, suggesting the absence of intra-genomic recombination events.

The phenetic analysis of the V3 region shows a significant amino acid stability in 9 residues of the V3 loop, for B-subtype isolates, confirming the strong selection for specific sequences involved in strategic functions regarding the immune response as well as cellular tropism and transmission. The different HIV-1 isolates identified in the current study show the subtype-specific tetrameric sequence at the apex of the V3 loop (GPGR for B clade, GPGQ for non-B clades), which is considered the target for the anti-V3 neutralizing antibodies. However, less-represented sequences have been identified at the tip of the B clade isolates (GPGG, GPGS, GPGQ), suggesting a constant diversification in this clade.

The overall results suggest that the B subtype is still largely predominant in the HIV-1 epidemic in Italy and is circulating among all risk groups. On the contrary, HIV-1 non-B subtypes in Italy are strictly associated with the heterosexual transmission and are identified in infections acquired in the last period (2001–2005). The three non-B-subtype HIV-1 isolates are strictly associated with heterosexual transmission. The samples PR06.03 and PR06.07, corresponding to an Ethiopian and a Nigerian subject respectively, belong to the subtype/CRF predominant in their respective Country of origin, suggesting an infection either prior to the immigration to Italy or subsequent but through heterosexual contacts with HIV-infected subjects from those Countries. The sample NA05.05, on the contrary, corresponds to an Italian-born subject with a possible partner/s from Regions with high endemicity for HIV infections.

These results confirm that in Italy, as in other Western European countries, non-B subtypes or recombinant forms are introduced by immigrants/migrants and transmitted at a low rate to the indigenous population. This would also explain the lower prevalence of non-B subtypes in Italy compared with other European countries with an older tradition of immigration waves and much tighter historic and economic links with African countries.

The presented data are representative of a nationwide molecular survey and, regardless the small sample size, are part of recurrent studies giving a constant updated picture of the genetic evolution of the HIV-1 epidemics in different risk groups in Italy.

## Competing interests

The authors declare that they have no competing interests.

## Authors' contributions

LB supervised the molecular and phylogenetic analysis and wrote the paper; AP and MT conducted molecular and phylogenetic analysis; FV and MCR enrolled patients and collected samples in Bologna; EP and CC enrolled patients and collected samples in Parma; CS and OP enrolled patients and collected samples in Naples; MLT contributed to the phylogenetic analysis and to writing the paper; FMB supervised the whole project. All authors read and approved the final manuscript.
